# Catalytic Activity of Water-Soluble Palladium Nanoparticles with Anionic and Cationic Capping Ligands for Reduction, Oxidation, and C-C Coupling Reactions in Water

**DOI:** 10.3390/nano15050405

**Published:** 2025-03-06

**Authors:** Jan W. Farag, Ragaa Khalil, Edwin Avila, Young-Seok Shon

**Affiliations:** Department of Chemistry and Biochemistry, California State University Long Beach, 1250 Bellflower Blvd., Long Beach, CA 90840, USA

**Keywords:** nanoparticles, palladium, reduction, oxidation, coupling, catalysis, semi-heterogeneous, colloidal catalysis

## Abstract

The availability of water-soluble nanoparticles allows catalytic reactions to occur in highly desirable green environments. The catalytic activity and selectivity of water-soluble palladium nanoparticles capped with 6-(carboxylate)hexanethiolate (C6-PdNP) and 5-(trimethylammonio)pentanethiolate (C5-PdNP) were investigated for the reduction of 4-nitrophenol, the oxidation of α,β-conjugated aldehydes, and the C-C coupling of phenylboronic acid. The study showed that between the two PdNPs, C6-PdNP exhibits better catalytic activity for the reduction of 4-nitrophenol to 4-aminophenol in the presence of sodium borohydride and the selective oxidation of conjugated aldehydes to conjugated carboxylic acids. For the latter reaction, molecular hydrogen (H_2_) and H_2_O act as oxidants for the surface palladium atoms on PdNPs and conjugated aldehyde substrates, respectively. The results indicated that the competing addition activities of Pd-H and H_2_O toward the π-bond of different unsaturated substrates promote either reduction or oxidation reactions under mild conditions in organic solvent-free environments. In comparison, C5-PdNP exhibited higher catalytic activity for the C-C coupling of phenylboronic acid. Gas chromatography–mass spectrometry (GC-MS) was mainly used as an analytical technique to examine the products of catalytic reactions.

## 1. Introduction

One of the important issues associated with organic reactions arises from generating toxic solvent waste. Therefore, water’s non-flammable, non-toxic, and economical characteristics make it more desirable as a solvent for organic catalysis applications [1,2]. With their semi-heterogeneous characteristics, colloidal metal nanoparticles can benefit from the features of both homogeneous and heterogeneous systems. For example, they can be freely mixed or dispersed in aqueous or organic solvents permitting homogeneous reactions while retaining the effective separability of heterogeneous systems [3,4]. To employ water as a solvent for semi-heterogeneous catalytic reactions, the preparation of catalytic metal nanoparticles readily soluble in water is required [5,6,7]. The use of organic ligands [7], polymers [8], dendrimers [9], and surfactants [10] to passivate the metal nanoparticle core is a common strategy for producing water-soluble colloidal nanoparticles. Since densely packed ligands can poison the catalytic activity of metal nanoparticles, it is important to control the density of ligands and open active surface sites to generate reactive colloidal nanoparticle catalysts [11]. In addition, it is also critical to maintain good enough stability to limit metal nanoparticles from aggregating, which hinders their activity, during catalysis.

The modified Brust–Schiffrin method, known as the thiosulfate method, has been employed to synthesize the catalytically active alkanethiolate-capped Pd nanoparticles (PdNPs) [3,4]. These alternative ligands are essential for regulating the balance between activity and stability of PdNPs for two primary reasons. First, an extra sulfite moiety in the alkyl thiosulfate ligand is successfully removed from the surface creating small voids on the surface of PdNPs during synthesis. Second, the slower passivation kinetics of alkyl thiosulfate ligands, which results from the ionic characteristics of the surface reactive groups, prevents the formation of a fully passivated metal nanoparticle surface. These characteristics of alkyl thiosulfate ligands result in the formation of metal nanoparticles capped with alkanthiolate monolayers of less dense packing [11,12]. The resulting PdNPs have been employed in various catalysis applications, including the hydrogenation and isomerization reactions of different unsaturated substrates such as alkenes and allylic alcohols [13,14].

The catalytic performance of water-soluble 11-(carboxylate)undecanethiolate-capped PdNP (C11-PdNP) and C6-PdNP catalysts with their ionic tail groups interacting with the solvent medium was previously investigated [15]. The studies found that the water-soluble PdNPs exhibit distinct catalytic properties and selectivity toward the hydrogenation of allyl alcohol depending on the chain length, with the C6-PdNP showing high activity and selectivity toward the hydrogenation product in water [15]. When hydrophobic allylic alcohol was used as a substrate, the catalytic reactions took place in biphasic conditions which required larger amounts of C6-PdNP (10 mol%) to meet the necessary kinetic efficiency of the reactions [16]. The C6-PdNP catalyst could produce the hydrogenation product as the major product (73%) with the formation of some isomerization products (27%) after 24 h of reaction.

For the biphasic reaction of allyl benzene, the biphasic catalytic reaction using C5-PdNP led to the formation of 3-phenylpropanal as the major product [17]. The peculiar oxidation of allyl benzene proved that H_2_O was added to the reaction intermediate, and the addition reaction is regioselective, favoring the anti-Markovnikov addition of H_2_O to the C=C bond of allyl benzene. The aldehyde selectivity of C5-PdNP was 83% which was higher than that of C6-PdNPs (66%). Contrary to C5-PdNP, C6-PdNP produced a substantial quantity of additional oxidation products such as cinnamaldehyde and cinnamic acid [17]. These findings suggested that ligand engineering can significantly influence the catalytic properties of metal nanoparticles and provide a strategy for designing highly selective catalysts that are efficient for unique catalytic reactions. In addition, the potential use of C6-PdNP for the oxidation of conjugated aldehyde under mild conditions in an aqueous environment was implied.

This paper focuses on the catalytic investigation of various reactions proving the catalytic versatilities of water-soluble colloidal PdNPs that operate in pure water. The catalytic activity and the kinetic behavior of PdNPs with different thiolate ligands are evaluated for three completely different model reactions: the reduction of 4-nitrophenol [18,19,20], the selective oxidation of conjugated aldehydes [21,22,23], and the homocoupling reaction of phenylboronic acid [24,25,26]. 4-Nitrophenol and its derivatives produced from the production of pesticides, insecticides, and other pharmaceutical and petrochemical products are among the refractory water pollutants [27]. Therefore, there has been a lot of research interest in the reduction of 4-nitrophenol by different types of nanoparticles [28,29,30]. α,β-unsaturated carboxylic acids are essential compounds, as they are among the most valuable intermediates and precursors for chemical production and pharmaceuticals [31]. Even though the oxidation of aldehydes into carboxylic acids is generally regarded as a simple and exothermic reaction, the most-known methods for the preparation of unsaturated carboxylic acids from their aldehyde counterparts have been devised under severe and/or complex reaction conditions or required greater than equimolar quantities of oxidants, resulting in the production of an equimolar waste byproduct [21,32,33,34,35,36]. The homocoupling of phenylboronic acids, much like the Suzuki reaction [37,38,39,40,41], has been a popular avenue for generating biaryls. As a result, this reaction has been extensively investigated, particularly via nanomaterial-based catalysts [24,25,26]. Herein, we present the use of water-soluble alkanethiolate-capped PdNPs with different ligand functionalities for the three model reactions mentioned above. The catalytic results of 4-nitrophenol reduction and phenylboronic acid homocoupling reactions are also compared to those of other water-soluble PdNPs in the literature [26,29,42,43,44]. Using water as our solvent, this research capitalizes on the excellent solubility of our nanoparticles in this system and the facile product separation post-catalysis.

## 2. Materials and Methods

The following reagents were purchased from the indicated suppliers and used as received. Tetra n-octylammonium bromide (TOAB), sodium borohydride (NaBH_4_), potassium tetrachloropalladate (II) (K_2_PdCl_4_), ethanol, methanol, acetone, dichloromethane, chloroform, acetonitrile, 4-nitrophenol, crotonaldehyde, and toluene were obtained from Thermo Fisher Scientific, Hampton, NH, USA. 6-Bromohexanoic acid (C_6_H_11_O_2_Br), (5-bromopentyl)trimethylammonium bromide, sodium thiosulfate pentahydrate (Na_2_S_2_O_3_•5H_2_O), biphenyl, phenylboronic acid, trans-cinnamaldehyde, and potassium bicarbonate were obtained from Sigma-Aldrich, St. Louis, MO, USA. Ethanol, methanol, acetone, dichloromethane, chloroform, acetonitrile, and toluene were obtained from Fisher Scientific. Deuterium oxide (D_2_O) and chloroform-d (CDCl_3_) were purchased from Cambridge Isotope Laboratories, Inc (Tewksbury, MA, USA). Water was purified using a Millipore Sigma Simplicity Ultrapure Water System.

Synthesis of 6-(carboxylate)hexanethiolate-capped Pd nanoparticles (C6-PdNP). In a 25 mL beaker, 0.40 mmol (132 mg) of K_2_PdCl_4_ was dissolved in 12 mL nanopure water. In another beaker, 2.0 mmol (1.096 g) of tetra-N-octylammonium bromide (TOAB) was dissolved in 25 mL of toluene. Both solutions were mixed in a 100 mL flask with constant stirring till a dark orange color in the organic layer and a clear color in the aqueous layer was observed. The aqueous layer was discarded using a separatory funnel, and the organic layer was placed in a 500 mL RBF. In a 50 mL beaker, 0.80 mmol of sodium S-(6-carboxylate)hexyl thiosulfate was dissolved in 10 mL of 25% methanol. The solution was added to the organic layer followed by the addition of 2.0 mmol of TOAB. The reaction mixture was continuously stirred for 15 min. In a separate 20 mL beaker, 8.0 mmol of NaBH_4_ was dissolved in 7 mL of nanopure water and vortexed for ~15 s. The sodium borohydride solution was delivered to the reaction flask dropwise over 60 s. The reaction solution darkens immediately after adding the sodium borohydride solution. After stirring the solution for 3 h, the organic layer was discarded using a separatory funnel and the solvent was removed using rotary evaporation. The dried product was suspended in 5 mL of methanol and centrifuged at 4900 RPM for washing and separation 3 times. The washed product was dissolved in water and dialyzed overnight, and the water was removed using rotary evaporation after dialysis. The dried nanoparticles were collected in a vial and stored in a vacuum.

Synthesis of 5-(trimethylammonio)pentanethiolate-capped Pd nanoparticles (C5-PdNP). In a 50 mL beaker, 0.40 mmol of K_2_PdCl_4_ was dissolved in 12 mL of nanopure water. A 2.0 mmol of TOAB was dissolved in 25 mL of toluene. Both solutions were mixed in a 100 mL flask with constant stirring until the color of the solutions changed to dark orange in the organic layer and a clear color in the aqueous layer. The aqueous layer was discarded using a separatory funnel, and the organic layer was placed in a 500 mL RBF. A 0.80 mmol of sodium S-(5-trimethylammonio)pentyl thiosulfate was dissolved in 10 mL of 25% methanol in a 50 mL beaker. The thiosulfate solution was added to the organic layer followed by the addition of 2.0 mmol of TOAB. The reaction mixture was continuously stirred for 15 min. In the separated 20 mL beaker, 8.0 mmol of NaBH_4_ was dissolved in 7 mL of nanopure water and vortexed for ~15 s. The sodium borohydride solution was delivered to the reaction flask dropwise over 60 s. The reaction solution darkens immediately after adding the sodium borohydride solution. The solution was stirred for 3 h and then the organic layer was discarded using a separatory funnel. The solvent was removed using rotary evaporation and the dried product was partially suspended in 25 mL acetonitrile/H_2_O (90:10). The suspended solution was again centrifuged at 4900 RPM for washing and separation, and the process was repeated 3 times. The washed product was dissolved in water and dialyzed overnight. The water was removed using rotary evaporation after dialysis. The dried nanoparticles were collected in a vial and stored under a vacuum

Reduction of 4-nitrophenol. Catalytic reduction of 4-nitrophenol by sodium borohydride follows the following steps: In a 10 mm path-length quartz cuvette, 2.5 mL of freshly prepared 0.1 M NaBH_4_ was mixed with 50 μL of a 1 × 10^−2^ M 4-nitrophenol solution. After thoroughly mixing the solution, 25 μL (0.1 mol%) of one of the two PdNP solutions was added. The reaction was monitored by obtaining UV-vis spectra from 200 nm to 500 nm in controlled time intervals. The 4-nitrophenolate ion was observed at λ_max_ near 400 nm. The reduction of 4-nitrophenol (2 mM in PBS) was monitored on SHIMADZU UV-2401PC UV-vis spectrophotometer.

Oxidation of α,β-unsaturated aldehydes. Catalysis reactions were set up by first dissolving 5 mol% of PdNP in 2.5 mL water in a 50 mL round-bottom flask. Hydrogen gas was purged for 15 min before 28 μL of a substrate, crotonaldehyde, was added to the reaction mixture. The reaction mixture was stirred at 1500 rpm with a magnetic stirrer at 20–22 °C for 24 h. The products were extracted with 3 mL of ethyl acetate. The solution containing the extracted products was filtered through silica gel to remove residual PdNP. The final solution was transferred to a GC vial for analysis. Crotonaldehyde and cinnamaldehyde are two different α,β-unsaturated aldehydes examined following the same procedure to study catalytic oxidation to the corresponding carboxylic acids.

Homocoupling of phenylboronic acid. Catalysis experiments were performed by adding 5 or 10 mol% of PdNP catalyst dissolved in nanopure water into a 50 mL RBF equipped with a stir bar. A 39.5 mg (0.323 mmol) of phenylboronic acid and 120 mg (0.868 mmol) of anhydrous potassium carbonate were added to the reaction flask. A stoichiometric amount of pyrene was used as an internal standard. The reaction was continuously stirred at room temperature for 24 h. The resulting products were analyzed via ^1^H NMR and/or GC-MS.

## 3. Results and Discussion

### 3.1. Characterization of Pd Nanoparticles

^1^H NMR was used to verify the integrity and purity of the ligand precursors, sodium S-(6-carboxylate)hexyl thiosulfate (Figure S1), and sodium S-(5-trimethylammonio)pentyl thiosulfate (Figure S2). The ^1^H NMR spectra of 6-(carboxylate)hexanethiolate-capped Pd nanoparticle (C6-PdNP) and 5-(trimethylammonio)pentanethiolate-capped Pd nanoparticle (C5-PdNP) are shown in Figures S3 and S4, respectively, which confirmed the successful synthesis of both water-soluble PdNPs. The UV-vis spectra of C6-PdNP and C5-PdNP shown in Figure S5 indicated the absence of the absorbances of Pd^2+^ at 406 nm and 309 nm, confirming the successful reduction into Pd^0^ [17]. The spectra of both PdNPs show an exponential absorbance decay from ca. 300 nm to 700 nm due to the Mie-scattering for spherical nanoparticles.

**Figure 1:**
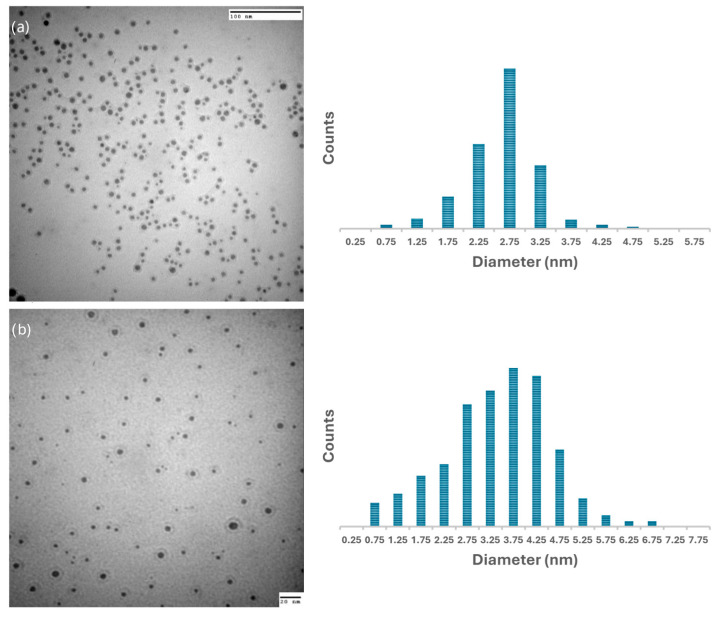
TEM images and histograms of (**a**) C6-PdNP and (**b**) C5-PdNP.

TEM images of C6-PdNP and C5-PdNP were obtained as shown in Figure 1. Both PdNPs are spherical shapes and free of any aggregate formation. The histogram analysis shows that the average core size of C6-PdNP is 2.63 ± 0.58 nm while that of C5-PdNP is 3.40 ± 1.13 nm.

Thermogravimetric analysis (TGA) results indicated that C6-PdNP contains approximately 74% Pd and 26% ligand, while C5-PdNPs have a Pd-to-ligand ratio of 67% Pd and 33% ligand (Figure S6). The values obtained from TGA are used to obtain actual Pd mass in C6-PdNP and C5-PdNP for the catalytic reactions.

### 3.2. Catalytic Reactions of 4-Nitrophenol

**Scheme 1:**
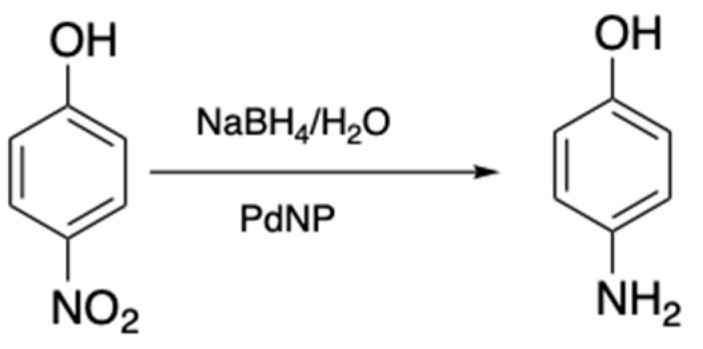
4-Nitrophenol reduction using NaBH_4_ with C6-PdNP or C5-PdNP.

The reduction of 4-nitrophenol by sodium borohydride in the presence of PdNP is used as a model reaction to study the effectiveness of PdNPs with different functional groups, carboxylate (C6-PdNP) and ammonium (C5-PdNP), as a catalyst as shown in Scheme 1. The well-established mechanism and description of this reaction by multiple authors make this model reaction a valuable tool for investigations in chemical kinetics [28,29,30,45].

**Figure 2:**
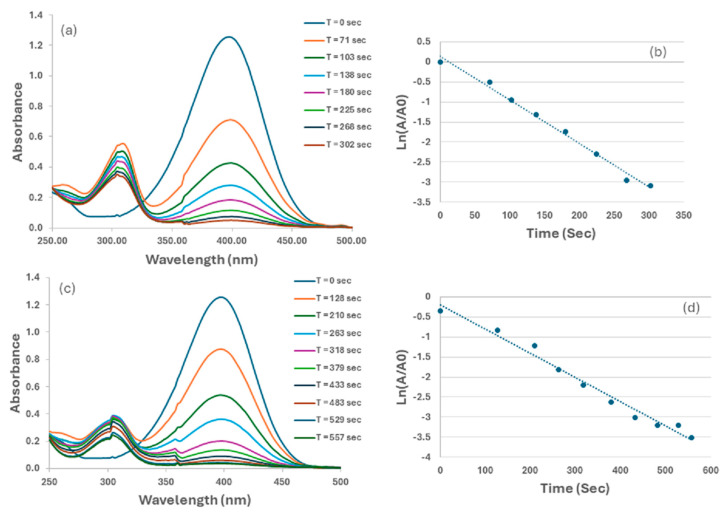
(**a**) UV–vis spectra for the catalytic reduction of 4-nitrophenol using C6-PdNP. (**b**) Absorbance dependence of the time for the reduction of 4-nitrophenol. (Conditions: [4-Nitrophenol] = 1 × 10^−2^ M; [NaBH_4_] = 0.1 M; [Pd] = 0.1 mol% Pd). (k_app_ = 1.09 × 10^−2^ S^−1^). (**c**) UV–vis spectra for the catalytic reduction of 4-nitrophenol using C5-PdNP. (**d**) Absorption dependence of the time for the reduction of 4-nitrophenol. (Conditions: [4-nitrophenol] = 1 × 10^−2^ M; [NaBH_4_] = 0.1 M; [Pd] = 0.1 mol% Pd). (k_app_ = 6.04 × 10^−3^ S^−1^).

During the 4-nitrophenol (4-NP) reduction to 4-aminophenol (4-AP), the real-time reaction, as shown in Figure 2a,c, was monitored by UV-vis spectroscopy. The observation of the absorbance decrease at 400 nm indicates a reduction reaction of 4-nitrophenoxide. Much research has shown that the reaction is first-order relevant to the concentration and absorbance of 4-nitrophenol when NaBH_4_ is in excess [46,47,48]. The rate constant, k_app_, was obtained from the linear slope of plotting [−ln(A/A_0_)] versus time in seconds (Figure 2b,d) where A is the absorbance in real-time and A_0_ is the initial absorbance. The comparison of the k_app_ values from the two PdNPs with different ligands indicates their relative catalytic activity performance. UV-vis spectra also confirmed the formation of 4-aminophenoxide which has an absorbance at ~300 nm. The produced 4-aminophenoxide was presumed to adsorb on the surface of Pd nanoparticles because amine-terminated ligands have relatively strong interactions with the Pd nanoparticle surface. This should explain why the absorbance bands at ~300 nm decrease after the initial appearance after the reduction of nitro groups. The immediate formation of 4-aminophenoxide as a major product with the reduction of 4-nitrophenoxide indicated the effectiveness of PdNP as the reduction catalyst.

The reaction started as soon as the PdNP was added to the reaction, with no induction time observed. This was evidenced by the linearity of ln(A/A_o_) versus time in seconds in Figure 2. The apparent kinetic rate constant (k_app_) was calculated from linear regression using the following Equation (1):

ln(A/A_o_)/t = k_app_ (1)

The summary results of the kinetics of PdNPs indicated that C6-PdNP (K_app_ = 1.09 × 10^−2^ S^−1^) has a higher catalytic activity than C5-PdNP (K_app_ = 6.04 × 10^−3^ S^−1^). Despite the potential electrostatic repulsion of the nitro group to the negatively charged C6-PdNP, the reactions’ kinetics still followed the expected trends corresponding to the relatively lower surface ligand density and slightly smaller average nanoparticle size of C6-PdNP. Other in situ-generated water-soluble PdNP catalysts, borohydride-stabilized PdNPs [29,42] and polyester dendrimer-supported PdNPs [43], were reported for the reduction of 4-nitrophenol in the presence of NaBH_4_. Their catalytic activities are compared by calculating the turn-over frequency (TOF) of each catalyst based on the Pd mol used for NP synthesis, the 4-nitrophenol mol used for the reaction, and the reaction time (min). Directly comparing their catalytic activities among different NP catalysts is difficult because each reaction is performed under different conditions (e.g., pH or concentration) and the NPs are in varying sizes (different fractions of surface atoms). At least the TOF comparisons among the Pd mol used allow the approximation of material usage efficiency. The TOFs of borohydride-stabilized PdNPs are 11.2 min^−1^ [29] and 0.02 min^−1^ [42], while that of polyester dendrimer-supported PdNPs is 24 min^−1^ [43]. In comparison, the TOF of C6-PdNP is 2 min^−1^ which is relatively comparable to those of other PdNPs despite the decreased proportion of active surface atoms due to the presence of thiolate ligands on the surface of PdNPs. Thiolate ligands allow C6- and C5-PdNPs to be isolated and stored in air before the catalysis use.

### 3.3. Catalytic Oxidation of Various α,β-Unsaturated Aldehydes

**Scheme 2:**
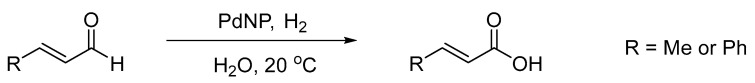
Catalytic oxidation of crotonaldehyde and cinnamaldehyde using water-soluble C6-PdNP and H_2_.

The two α,β-unsaturated aldehydes were examined to study the potential of PdNP for the catalytic oxidation of conjugated aldehyde to the corresponding unsaturated carboxylic acids under mild conditions (Scheme 2). The standard catalytic reactions were attempted with 5 mol% of PdNP at room temperature under the atmospheric pressure of H_2_ gas in water.

The reaction of cinnamaldehyde was first studied to confirm the capability of PdNP as the oxidation catalyst of α,β-unsaturated aldehyde in water (Table 1). Since the double bond has highly delocalized π electrons and, consequently, a greater overlap distance, it is likely to react with soft electrophiles such as hydrogen. In contrast, it is presumed that the second electrophilic site, carbonyl carbon, is more likely to react with hard nucleophiles like H_2_O. The catalytic reactions of cinnamaldehyde resulted in a 12% conversion of cinnamaldehyde to both the oxidation and hydrogenation products such as cinnamic acid, 3-phenylpropanoic acid, and 3-phenylpropanal by C6-PdNP after 24 h (Figures S7–S10). Among these three products, the major product was cinnamic acid with 60% selectivity. 3-Phenylpropanal and 3-phenylpropanoic acid are also identified with 28% and 12% selectivities, respectively. The results indicated that the overall reaction is noticeably slow due to the poor solubility of cinnamaldehyde in the solvent medium, water, and the steric interference caused by the interactions of cinnamaldehyde with the surface ligand on PdNPs. The selectivity toward oxidation was relatively high, but the reaction toward the hydrogenation of C=C still took place. This indicates that a conjugating benzene ring at the *β* C from the carbonyl group slightly hinders the oxidation reactivity of α,β-unsaturated aldehyde. In comparison, the catalytic reaction of cinnamaldehyde using C5-PdNP did not produce cinnamic acid at all and instead generated the hydrogenation product in low yields under the same conditions. This result suggested that the difference in catalytic selectivity of C6-PdNP and C5-PdNP might arise from the ligand characteristics of an anionic functional group in C6-PdNP.

The activity of water-soluble crotonaldehyde, which produces crotonic acid with a selective oxidation reaction, was also examined. The absence of delocalization of π electrons by the benzene ring in crotonaldehyde, in addition to its different solubility, was of interest. Crotonaldehyde reacted for 6 h in the presence of C6-PdNP. The GC spectrum shown in Figure S11 indicates the presence of a broad peak at the retention time of 5.31–5.59 min for crotonic acid produced by the oxidation of the aldehyde functional group. No other significant peaks were observed in the GC spectrum and the % selectivity of crotonic acid was estimated to be >95%. The MS spectrum in Figure S12 confirmed the identity of crotonic acid matching with the reference MS data. Besides the solvent peak, small unidentified peaks appear between 3.90 and 4.30 min with less than 5% selectivity. This catalysis result indicated the high selectivity for the oxidation of the aldehyde group in the presence of a reactive C=C bond for small and water-soluble α,β-unsaturated aldehydes using this simple and environmentally friendly process.

**Scheme 3:**
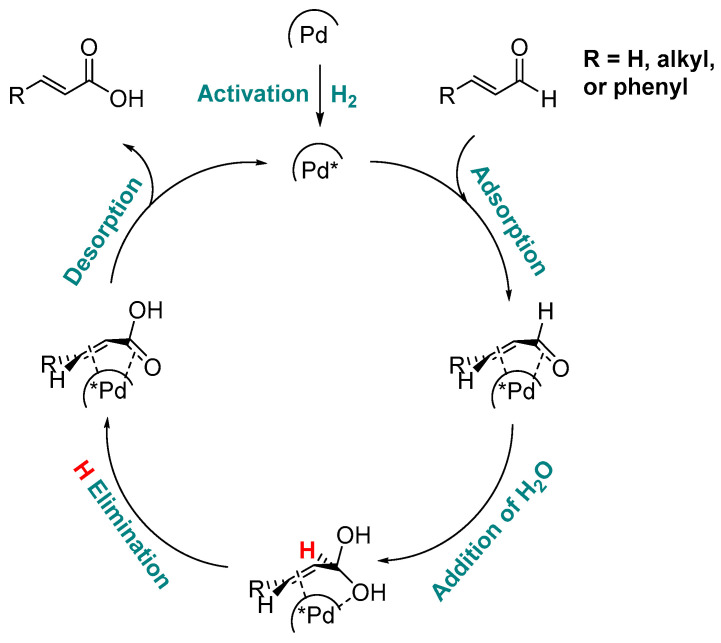
Proposed mechanism of selective oxidation of conjugated aldehyde.

Scheme 3 shows the proposed mechanism for the oxidation of α,β-unsaturated aldehydes to the corresponding carboxylic acids. The synthesis of crotonic acid from crotonaldehyde works only in the presence of H_2_ gas as evidenced by the results in Table 1 (iv). The oxidation reaction starts with the adsorption of hydrogen (H_2_) molecules onto the surface of the Pd nanoparticles as reported in the previous work [17]. The Pd **activation** process involves the dissociation of H_2_ into two hydride atoms (2H^−^) on the catalyst surface with the formation of oxidized Pd ions that can initiate the catalytic reaction. The activated PdNP allows the facile **adsorption** of crotonaldehyde, makes crotonaldehyde more electrophilic, and promotes H_2_O to undergo nucleophilic **addition** reaction to the carbonyl. In the catalytic cycle, therefore, H_2_O acts as a main oxidation reagent of the aldehyde functional group. This process results in the formation of 1,1-geminal diol-like structures known as hydrates. The hydrates are not stable enough to be isolated as the equilibrium shifts back to starting materials upon isolation (due to Le Chatelier’s principle). However, hydrate-like structures are the reactive species in the aqueous oxidation of aldehydes to carboxylic acids. The subsequent reductive **elimination of H** on the hydrate carbon, which is one of the key steps required for the oxidation of aldehyde to carboxylic acid, completes the formation of the carboxylic acid functional group. The negatively charged ligand-capped C6-PdNP might play a role (base) in promoting the removal of H from hydrate OH during the oxidation process. The **desorption** of crotonic acid from the Pd nanoparticle surface completes the catalytic cycle. Despite the presence of Pd-H, the addition of hydrogen atoms to the C=C double bond in crotonaldehyde was not favored. This is in part due to the low solubility of H_2_ in water limiting the amount of Pd-H on the nanoparticle surface, especially under atmospheric pressure and with a small volume of added H_2_ gas.

These results confirmed that the selective oxidation of α,β-unsaturated aldehydes to the corresponding α,β-unsaturated carboxylic acids can be achieved by using hydrogen gas and water as oxidants for surface Pd and the aldehyde, respectively, with water-soluble Pd nanoparticles as a catalyst. The C6-PdNP with carboxylate functional group could effectively oxidize small water-soluble and unhindered α,β-unsaturated aldehydes such as crotonaldehyde under mild and environmentally friendly organic solvent-free conditions.

### 3.4. Homocoupling of Phenylboronic Acid

**Scheme 4:**
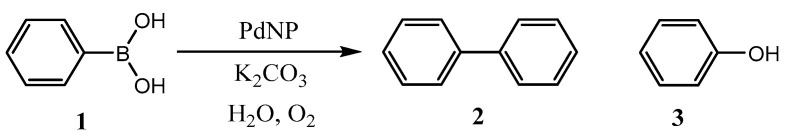
Catalytic C-C coupling reaction of phenylboronic acid using water-soluble PdNPs in water.

The catalytic activities of water-soluble alkanethiolate-capped PdNPs with different ligand functionalities are investigated for the homocoupling of phenylboronic acids (1) (Scheme 4) [49,50]. This reaction has been extensively studied, particularly for nanomaterial-based catalysts [24,25,26,44,50]. Catalysis assays were performed using 5 or 10 mol% PdNPs dissolved in H_2_O with or without a base as depicted below in Table 2. Following the traditional reaction conditions for the homocoupling of phenylboronic acids, a base (potassium carbonate) was used to increase the nucleophilicity of the organoboron substrate to promote transmetallation [49,50]. Entry i depicts the best attempt to produce biphenyl with the C6-PdNP, which was predicted to be the most effective PdNP for this reaction, particularly because of their high activity toward two previous catalytic reactions. However, even with 10 mol% of the C6-PdNP, the catalytic reaction could only produce a 25% yield of biphenyl (2) with a minute amount of the minor product, phenol (3, 4%). This result indicated the low catalytic reactivity of C6-PdNP for the homocoupling reaction.

For the catalytic homocoupling reaction with C5-PdNP, using the 3 equivalents of the base (entry ii) resulted in an 85% yield of biphenyl utilizing 5 mol% catalyst. When the reaction was performed with a reduced base (1.5 equivalents, entry iii), the reaction resulted in only a 29% yield of biphenyl. Excluding the PdNP catalyst entirely (entry iv) led to the formation of no coupling product. These results indicated the potential of cationic ligand-capped C5-PdNP for an appreciable degree of transmetallation with the base presence. The large difference in the catalytic activity between C5-PdNP and C6-PdNP may stem from the electrostatic repulsion of phenylboronate from the anionic carboxylate functionalized ligands on C6-PdNP. The higher activity of C5-PdNP for the coupling of phenylboronic acid indicated the ligand’s functionality difference could have a more significant effect than the core size and the surface ligand density of PdNP. TOF of C5-PdNP for the homocoupling reaction in pure H_2_O was 0.71 h^−1^ based on the Pd mol. In comparison, polyvinylpyrrolidone (PVP)-stabilized Pd nanocubes catalyzed the homocoupling reaction in a biphasic system of water and methylcyclohexane at 80 °C with a TOF of ~20 h^−1^ [26]. PdNP colloids formed upon degradation of a palladacyclic complex could catalyze aryl boronic acid in H_2_O-CH_3_CN cosolvent at 80 °C with a TOF of ~11 h^−1^ [44]. These colloidal PdNP catalysts employed for homocoupling reactions exhibited higher catalytic activity. Still, they required an organic solvent as a co-solvent and an elevated temperature for successful reactions.

## 4. Conclusions

This paper showed that the core size, surface ligand density, and ligands with different functional groups influence the catalytic activity of Pd nanoparticles in water. Specifically, the C6-PdNP with carboxylate functional group exhibited higher catalytic activity for 4-nitrophenol reduction in the presence of sodium borohydride due to its smaller average core size and lower surface ligand density. The C6-PdNP also showed its efficiency in the oxidation of α,β-unsaturated aldehydes, such as crotonaldehyde and cinnamaldehyde, to the corresponding α,β-unsaturated carboxylic acids requiring only hydrogen gas in water and room temperature. Hydrogen activates Pd nanoparticles by oxidizing surface Pd atoms and H_2_O acts as the oxidation reagent for α,β-unsaturated aldehydes. In comparison, the C5-PdNP with an ammonium functional group was found to have higher catalytic activity in the C-C coupling reaction of phenylboronic acid. The results proved that the characteristics of surface ligand functionalities also influence the overall catalytic activity for certain reactions, presumably via the repulsive interactions of the substrate and ligand of Pd nanoparticles. The catalytic studies in pure water also indicated that the overall scope of the substrate is currently limited to small and water-soluble reactants. Future studies will be attempted to expand the substrate scope for each model reaction beyond water-soluble reactants. The insights obtained from this study into the selectivity and catalytic activity of water-soluble Pd nanoparticles will be utilized to design more efficient green catalysts for various reactions in organic synthesis.

## Figures and Tables

**Table 1:** GC-MS results for the catalytic oxidation of cinnamaldehyde and crotonaldehyde in H_2_O using C6- and C5-PdNP (5 mol% Pd; 1 atm H_2_; Reaction Time: 6 (crotonaldehyde) or 24 h (cinnamaldehyde)).

			Selectivity (%) ^a^		
Entry	Catalyst	Substrate	RCH = CHCO_2_H	RCH_2_CH_2_CO_2_H	RCH_2_CH_2_CHO
i	C6-PdNP	cinnamaldehyde	7.2	3.4	1.4
ii	C5-PdNP	cinnamaldehyde	0	0	8
iii	C6-PdNP	crotonaldehyde	95 ^b^	-	-
iv	C6-PdNP (no H_2_)	crotonaldehyde	0	0	0

^a^ The reported values are the % selectivity rather than the actual % yield. ^b^ There are ~5% of unidentified products.

**Table 2:** NMR and GC-MS results for homocoupling phenylboronic acid in H_2_O using C5- and C6-PdNP (Reaction Time: 24 h).

		Yield (%)	
Entry	Catalyst	2	3
i	10 mol% C6-PdNP (3.0 eq)	25	4
ii	5 mol% C5-PdNP (3.0 eq)	85	15
iii	5 mol% C5-PdNP (1.5 eq)	29	8
iv	No catalyst (3.0 eq)	0	4

## Data Availability

The original contributions presented in this study are included in the article/Supplementary Materials. Further inquiries can be directed to the corresponding author(s).

## Supplementary Materials

The following supporting information can be downloaded at: https://www.mdpi.com/article/10.3390/nano15050405/s1. Synthesis of sodium S-(6-carboxylate)hexyl thiosulfate and sodium S-(5-trimethylammonio)pentyl thiosulfate ligands; instrumentations; characterization of sodium S-(6-carboxylate)hexyl thiosulfate and sodium S-(5-trimethylammonio)pentyl thiosulfate ligands: Figure S1: ^1^H NMR spectrum of sodium S-(6-carboxylate)hexyl thiosulfate ligand. Figure S2: ^1^H NMR spectrum of sodium S-(5-trimethylammonio)pentyl thiosulfate ligand; characterization of Pd nanoparticles synthesized by alkyl thiosulfate method. Figure S3: ^1^H NMR spectrum of 6-(carboxylate)-1-hexanethiolate-capped PdNP (C6-PdNP). Figure S4: ^1^H NMR spectrum of 5-(trimethylammonio)pentanethiolate-capped PdNP (C5-PdNP). Figure S5: UV-vis spectra of (a) C6-PdNP and (b) C5-PdNP; catalytic reactions of Pd nanoparticles. Figure S6: TGA of (a) C6-PdNP (carboxylate) and (b) C5-PdNP (ammonium). Figure S7: Gas chromatogram obtained after the reaction of cinnamaldehyde with C6-PdNP in the presence of hydrogen gas in water for 24 h (extracted with ethyl acetate and then passed through a pipette filled with silica gel). Figure S8: Mass spectrum of the peak at 12.45 min which corresponds to cinnamic acid. Figure S9: Mass spectrum of the peak at 10.38 min which corresponds to 3-phenylpropanal. Figure S10: Mass spectrum of the peak at 11.34 min which corresponds to cinnamaldehyde. Figure S11: Gas chromatogram obtained after the reaction of crotonaldehyde with C6-PdNP in water after 6 h (extracted with ethyl acetate and then passed through a pipette filled with silica gel). Figure S12: Mass spectrum of the broad peak at 5.31–5.59 min corresponds to crotonic acid.

## Abbreviations

The following abbreviations are used in this manuscript:

PdNP	Palladium nanoparticle
GC-MS	Gas chromatography-mass spectrometry
TOAB	Tetra n-octylammonium bromide
RBF	Round bottom flask
RPM	Revolutions per minute
PBS	Phosphate-buffered saline
NMR	Nuclear magnetic resonance
TEM	Transmission electron microscopy
TGA	Thermogravimetric analysis
4-NP	4-Nitrophenol
TOF	Turnover frequency
