# Supplementary material for: Catalytic Activity of Water-Soluble Palladium Nanoparticles with Anionic and Cationic Capping Ligands for Reduction, Oxidation, and C-C Coupling Reactions in Water
Source: Nanomaterials (Basel). 2025 Mar 6;15(5):405. doi: 10.3390/nano15050405 (PMC11901881; doi:10.3390/nano15050405)
Supplement: Supplementary file 1 [file nanomaterials-15-00405-s001.zip › nanomaterials-3486883-supplementary.pdf]

Supplementary Information (SI) For:

Catalytic Activity of Water-Soluble Palladium Nanoparticles with Anionic and Cationic Capping Ligands for Reduction, Oxidation, and C-C Coupling Reactions in Water

Jan W. Farag, Ragaa Khalil, Edwin Avila, and Young-Seok Shon*

Department of Chemistry and Biochemistry, California State University Long Beach, 1250 Bellflower Blvd., Long Beach, CA 90840, USA

For correspondence: Email: ys.shon@csulb.edu. Phone: 562-985-4466. Fax: 562-985-8547.

I. Synthesis of sodium S-(6-carboxylate)hexyl thiosulfate
II. Synthesis of sodium S-(5-trimethylammonio)pentyl thiosulfate
III. Instrumentations
IV. Characterization of thiosulfate ligands and Pd nanoparticles
V. Catalytic reactions of Pd nanoparticles

I. Synthesis of sodium S-(6-carboxylate)hexyl thiosulfate

The ligand precursor was prepared according to the previously published procedure.[1] A 25 mmol (4.87 g) of 6-bromohexanoic acid and 25 mmol (6.21 g) of sodium thiosulfate pentahydrate were dissolved in a mixture of solvents with 50 mL nanopure water and 50 mL ethanol in a 1000 mL round-bottom flask (RBF). The flask was refluxed for 3 hours with constant stirring. The mixture was then allowed to cool to room temperature and then the solvent was removed by a rotary evaporator. A 30 mL of preheated ethanol was added to dissolve the ligand. The dissolved ligand was subsequently filtered using a Buchner filtration set to remove solid impurities - 10 mL of preheated ethanol was added to the filter paper to prevent crystallization. The hot, filtered solution was cooled to room temperature and then deposited in an ice bath for one hour to accelerate crystallization. The crystalized products were isolated using vacuum filtration and then dried under vacuum. The final product was stored in a vial under nitrogen for future use.

II. Synthesis of sodium S-(5-trimethylammonio)pentyl thiosulfate

An 8.65 mmol of (5-bromopentyl)trimethylammonium bromide and 9.08 mmol of sodium thiosulfate pentahydrate were dissolved in 10 mL nanopure water in a 50 mL RBF. In an oil bath, the flask was refluxed for 6 hours with constant stirring. The mixture is then allowed to cool to room temperature overnight for slow precipitation while stirring gently. The next day, the precipitated ligand was further cooled by placing the flask that had the precipitated ligand in an ice bath for an hour and then filtered after washing it with nanopure water. The final white crystalline product was collected and stored under a vacuum.

III. Instrumentations

^{1}H NMR. ^{1}H NMR was conducted on a Bruker Avance-II 400 MHz at 298 K. ^{1}H NMR spectra of the ligands and PdNPs were analyzed using MestreNova software. D_2O was used as the

solvent for both ligands and PdNPs. Residual solvent peaks at 4.70 ppm and 7.26 ppm were used as internal references.

UV-Vis spectroscopy. The UV-Vis spectra of the synthesized PdNPs were obtained using a Shimadzu UV-2450 spectrometer with absorbance intensities measured from 300 nm to 900 nm. Nanopure water was used as the solvent. Data were analyzed using UVProbe software.

Thermogravimetric analysis (TGA). Thermogravimetric analysis (TGA) data was acquired on a Discovery Series TA SDT650 with ca. 4 mg PdNP in an alumina pan heated from room temperature to 600 °C with a flow rate of 100 mL/min of Argon gas and a temperature ramp rate of furnace set at 20 ºC/min. Data were analyzed via Trios software.

Transmission electron microscopy (TEM). TEM images were analyzed by using JEOL 1200 EX II electron microscope. The TEM sample was prepared by diluting PdNPs to 0.002 mg/mL in methanol solvent. The samples were then placed in a 200-mesh standard carbon-coated copper grid and allowed to dry for approximately 30 min. The images for the particle size distribution were analyzed by Scion Image Beta Release 4.0 or ImageJ.

Gas chromatography-mass spectrometry. Gas chromatography (GC) spectrum was acquired on a Thermo Scientific TRACE 1300 with a flow rate of 1.5 mL/min of helium. The temperature was kept at 30 °C for 5 min and then increased to 260 °C with a rate of 20 °C/min. The GC detector was an ISQ QD Single Quadruple Mass spectrometer. The front column of GC used was HP-5MS with a length of 30.00 m, a diameter of 0.250 mm, and a film thickness of 0.5 μm. The mobile phase was a stream of inert helium gas and the stationary phase was a capillary column for the high sensitivity required to separate the catalytic reaction products. The identity of each peak-generating compound was determined by an algorithm matching experimental data to a

spectral database. Chromeleon software was used to operate the GC-MS, analyze chromatograms, and identify peaks.

IV. Characterization of thiosulfate ligands and Pd nanoparticles

Characterizations of Water-Soluble Thiosulfate Ligands. ^{1}H NMR spectrum of sodium S-(6-carboxylate)hexyl thiosulfate dissolved in D_2O is illustrated in Figure S1. All five chemical shifts of the ligand are characteristic of sodium S-(6-carboxylate)hexyl thiosulfate.[1] The absence of any foreign peaks verifies that the synthesized ligand is free of any impurities including the precursor, 6-bromohexanoic acid. ^{1}H NMR (400 MHz, D_2O): δ 3.13 (t, 2H, C**H**$_2S_2O_3^-$), δ 2.42 (t, 2H, C**H**$_2$COO$^-$), δ 1.81 (m, 2H, C**H**$_2$), δ 1.579 (m, 2H, C**H**$_2$), and δ 1.48 (m, 2H, C**H**$_2$).

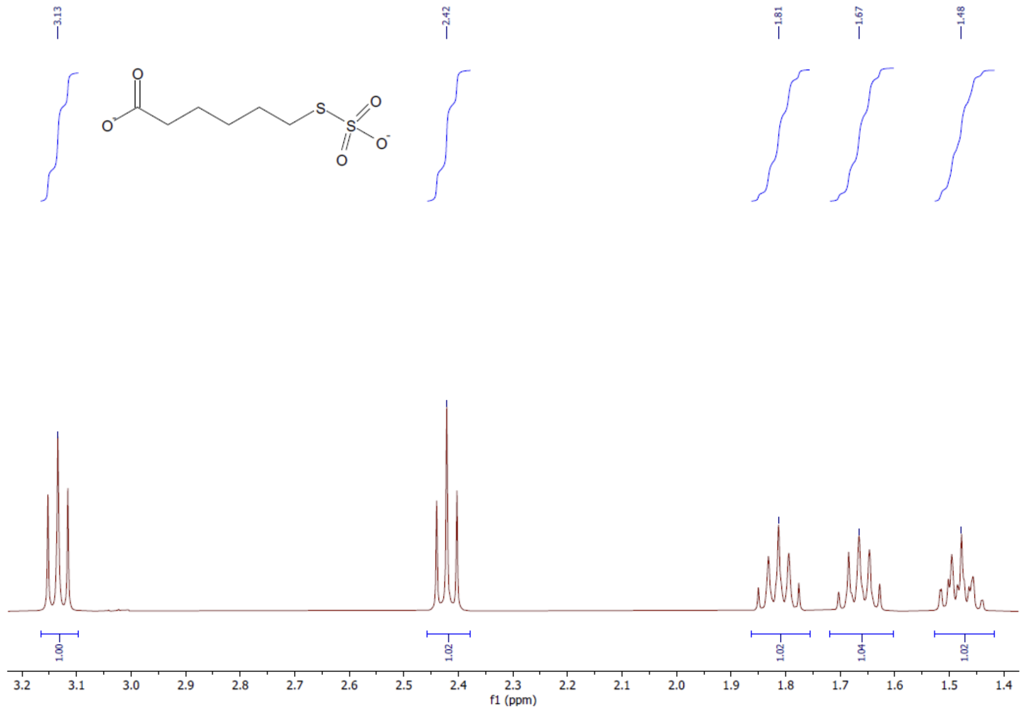

Figure S1. ^{1}H NMR spectrum of sodium S-(6-carboxylate)hexyl thiosulfate ligand.

The ^{1}H NMR spectrum of sodium S-(5-trimethylammonio)pentyl thiosulfate ligand is shown in Figure S2.[2] All the chemical shifts are characteristic of the ligand and the absence of

any additional chemical shifts indicate that the synthesized ligand is in a pure form. It was observed from the ^{1}H NMR that there is an overlap for signals at δ 3.03 – 3.08. ^{1}H NMR (400 MHz, D_2O): δ 3.27 (t, 2H, C**H**$_2$N$(CH_3)_3$), δ 3.06 (t, 2H, C**H**$_2S_2O_3^-$), δ 3.05 (s, 9H, N(C**H**$_3$)$_3$, δ 1.79 (tt, 2H, C**H**$_2$), δ 1.79 (tt, 2H, C**H**$_2$), and δ 1.44 (tt, 2H, C**H**$_2$).[2]

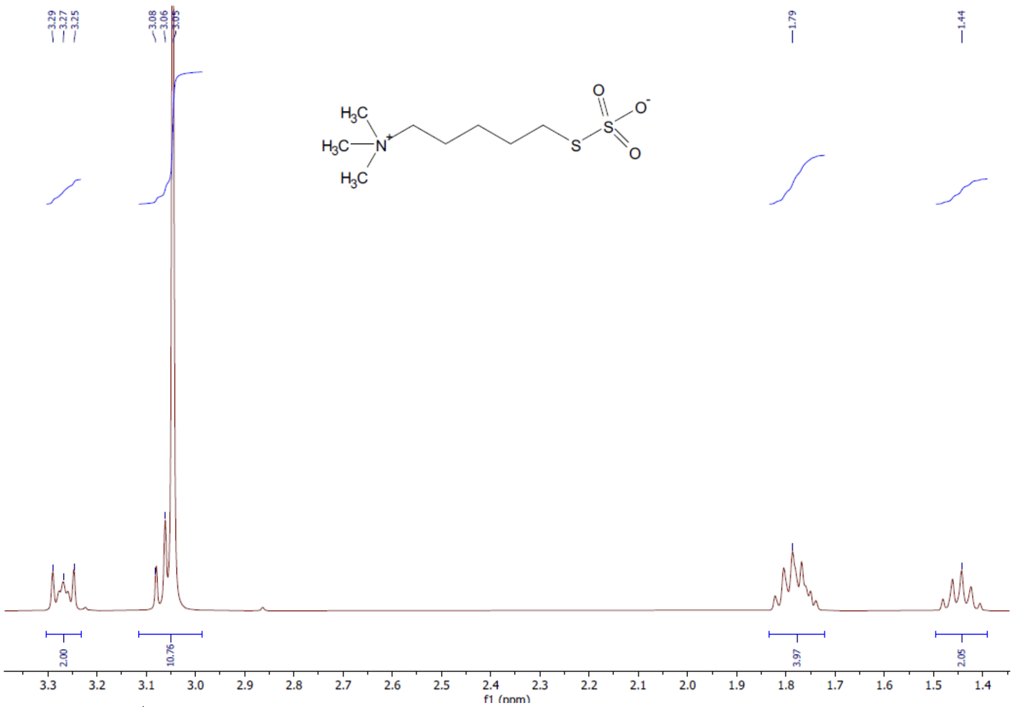

Figure S2. ^{1}H NMR spectrum of sodium S-(5-trimethylammonio)pentyl thiosulfate ligand.

Characterization of Pd nanoparticles. The ^{1}H NMR spectrum for the nanoparticles synthesized with the sodium S-(6-carboxylate)hexyl thiosulfate ligand is shown in Figure S3. Two broad chemical shifts at 1.54 ppm and 2.19 ppm observed in the spectrum represent 4 and ~2 hydrogens, respectively. The observed broad signals at 1.54 ppm represent the methylene hydrogens of the alkyl chain (δ and γ CH_2 to S). The broad signals at 2.19 ppm represent the hydrogens α to the carbonyl.[1] The absence of two methylene proton signals (α and β CH_2 signals to the S) confirms a covalent bonding between the thiolate group and the metal surface of the Pd. In addition, the absence of any sharp peaks explains the absence of any free ligands.

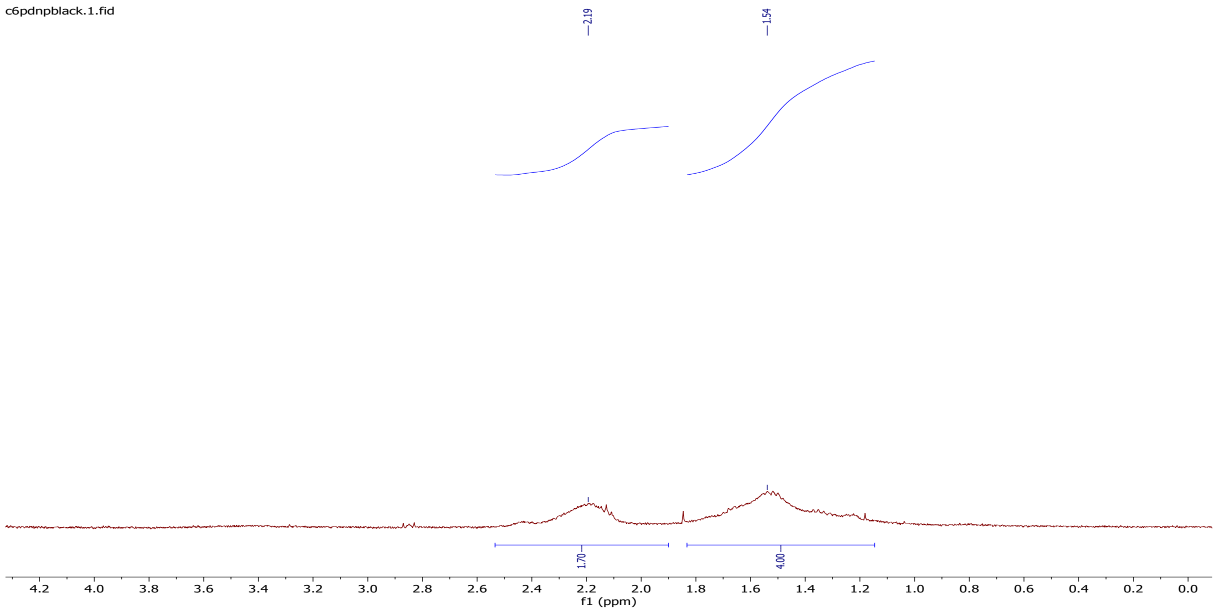

Figure S3. ^1H NMR spectrum of 6-(carboxylate)-1-hexanethiolate-capped PdNP (C6-PdNP).

The ^1H NMR spectrum for the nanoparticles synthesized with the S-(5-trimethylammonio)pentyl thiosulfate ligand is shown in Figure S4.[2] There are four total broad chemical shifts at 1.54 ppm, 1.80 ppm, 3.07 ppm, and 3.29 ppm were observed in the spectrum. The chemical shifts at 1.58 ppm and 1.80 ppm represent methylene hydrogens of the alkyl chain (CH_2 γ to S) and hydrogens *δ* to the thiolate group, respectively. The signals at 3.07 ppm show the 9 hydrogens surrounding the nitrogen, while the peaks at 3.29 ppm show α hydrogen methylene corresponding to the ammonium group. Since there is a covalent bonding between the thiolate group and the metal surface of the Pd, it explains the absence of two methylene proton signals (α and β CH_2 chemical shifts to the S).[2]

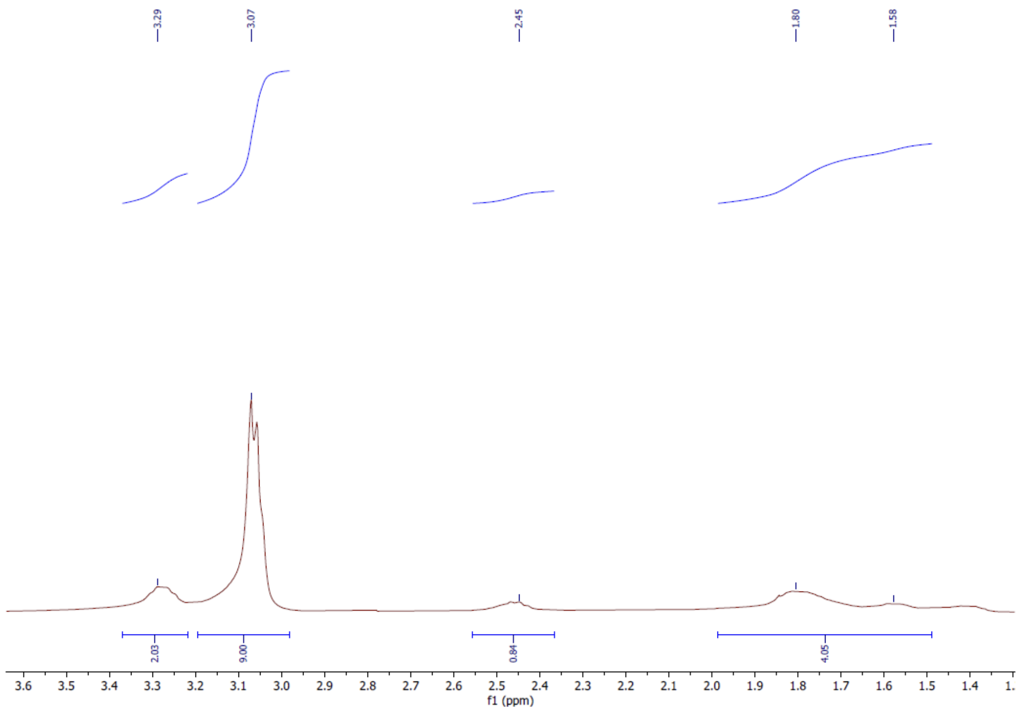

Figure S4. ^{1}H NMR spectrum of 5-(trimethylammonio)pentanethiolate-capped PdNP (C5-PdNP).

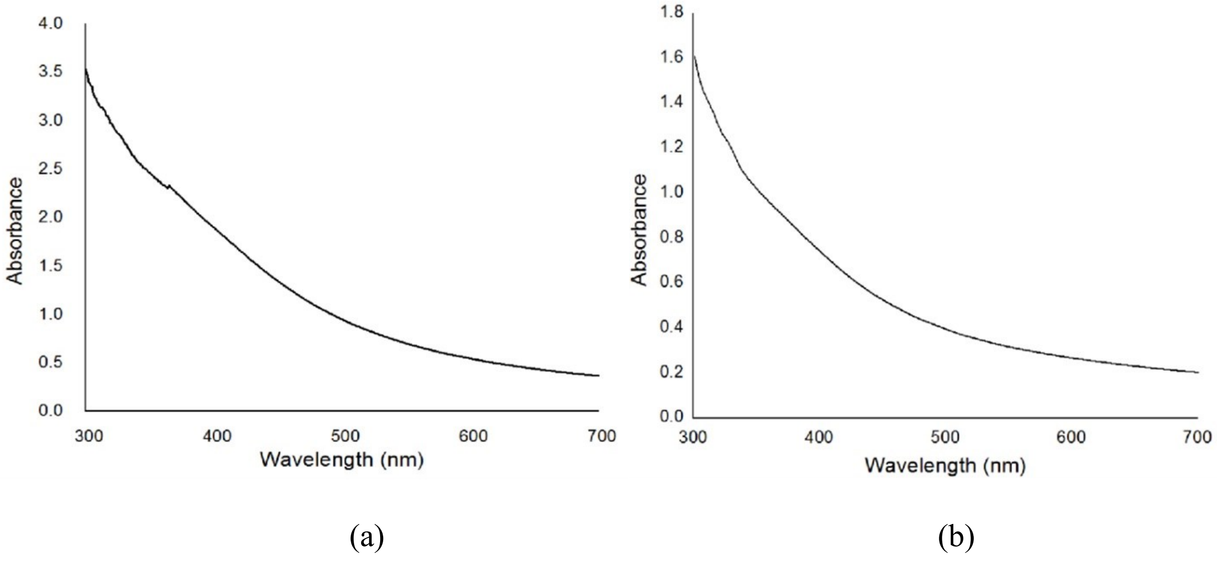

Figure S5. UV-Vis spectra of (a) C6-PdNP and (b) C5-PdNP.

Thermogravimetric analysis (TGA) results show that there is the period from room temperature to ca. 250 °C when the encapsulated solvent (water) evaporated. Because the synthesized PdNP dissolved in water contains ionic functional groups, it is inevitable for the PdNP to contain some extent of water even after extensive drying at near room temperature. From ca. >250 °C the ligand started to detach from the particle surface and the mass of PdNP metal became constant at around >550 °C.

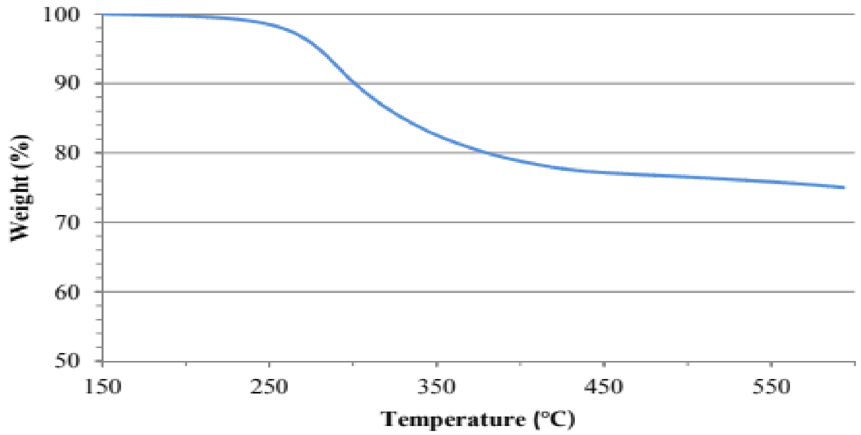

(a)

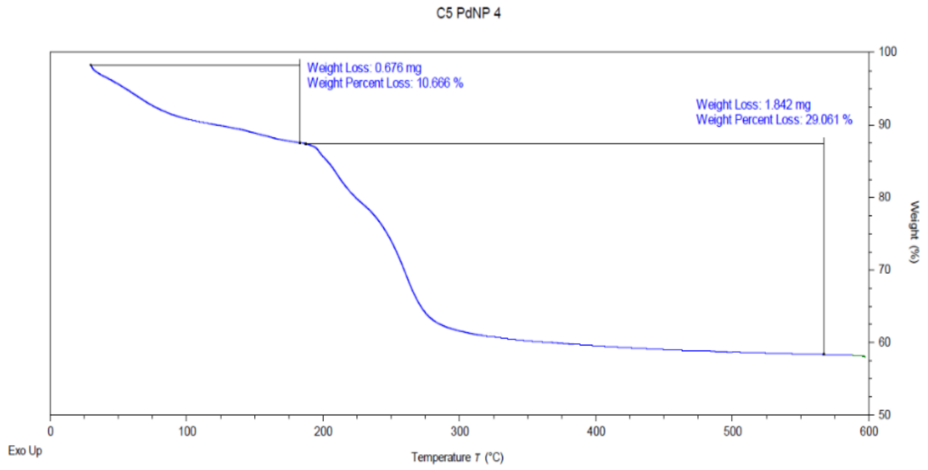

(b)

Figure S6. TGA of (a) C6-PdNP (carboxylate) and (b) C5-PdNP (ammonium).

V. Catalytic reactions of Pd nanoparticles

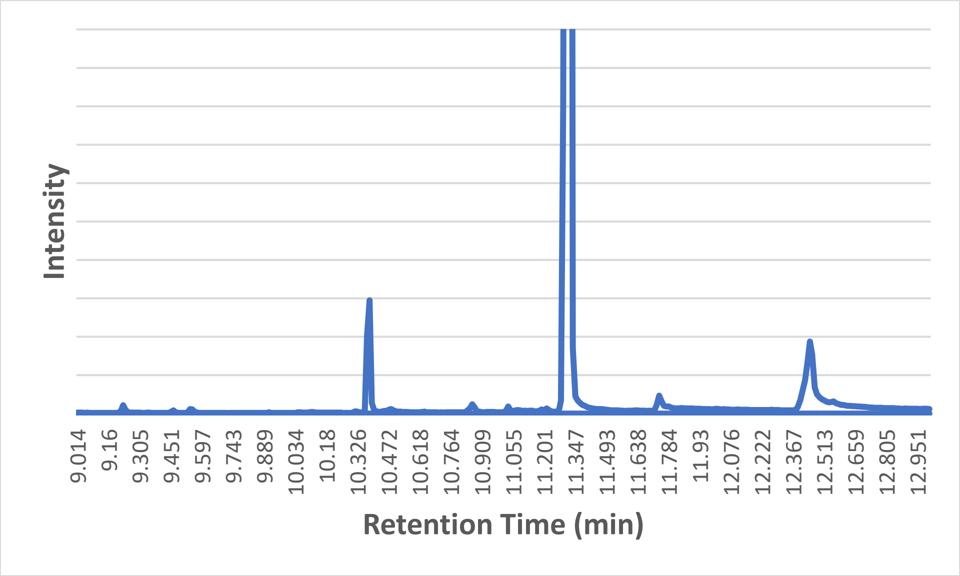

Figure S7. Gas chromatogram obtained after the reaction of cinnamaldehyde with C6-PdNP in the presence of hydrogen gas in water for 24 h (extracted with ethyl acetate and then passed through a pipette filled with silica gel).

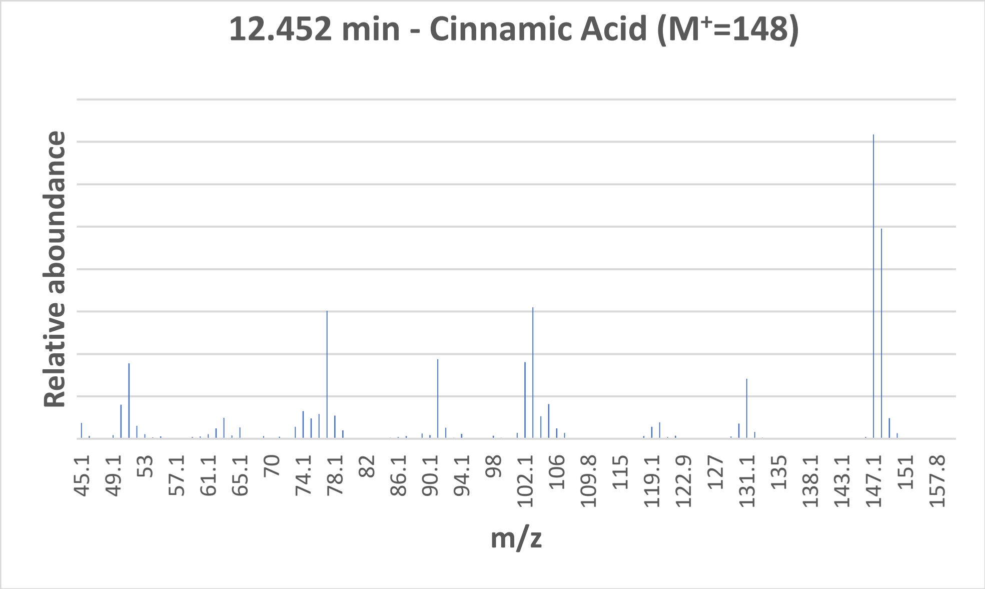

Figure S8. Mass spectrum of the peak at 12.45 min which corresponds to cinnamic acid.

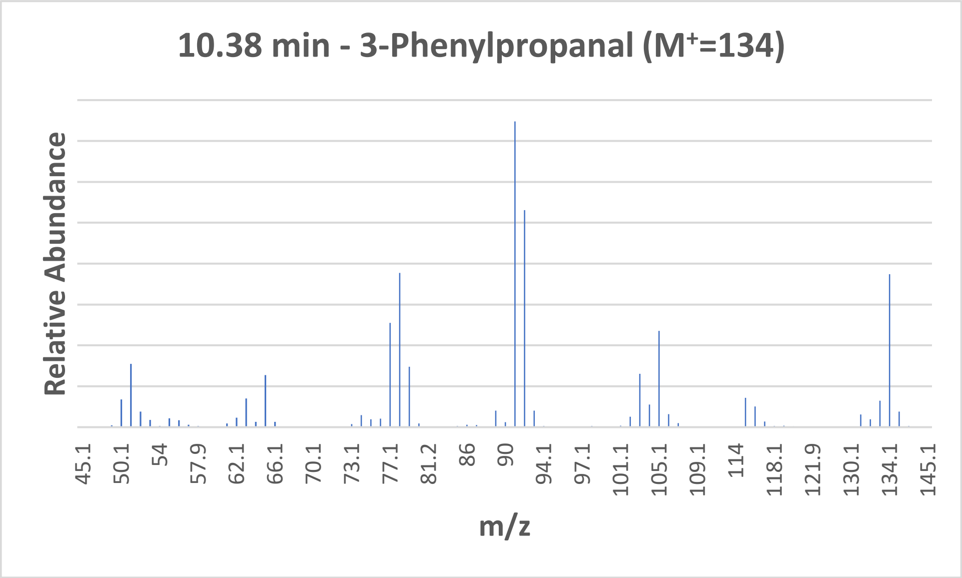

Figure S9. Mass spectrum of the peak at 10.38 min which corresponds to 3-phenylpropanal.

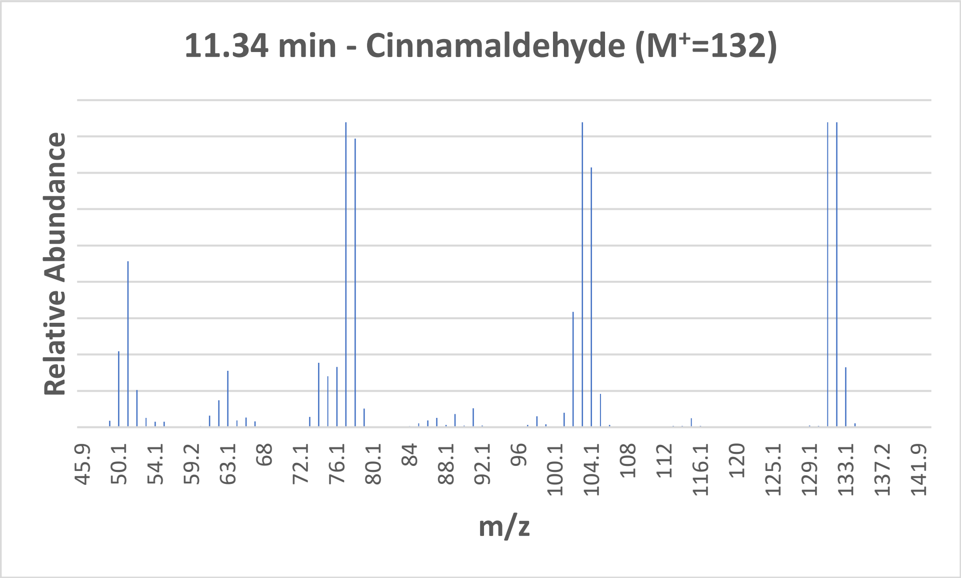

Figure S10. Mass spectrum of the peak at 11.34 min which corresponds to cinnamaldehyde.

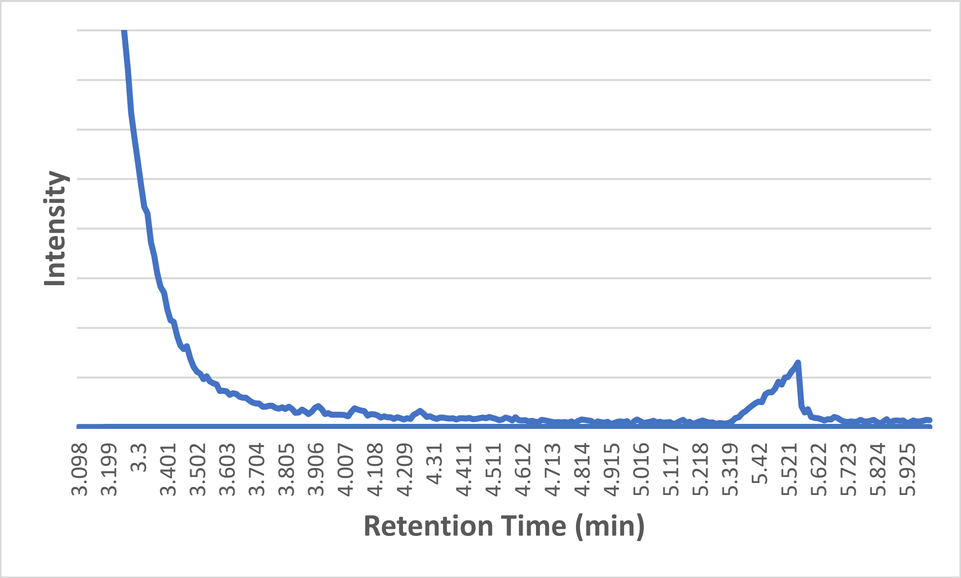

Figure S11. Gas chromatogram obtained after the reaction of crotonaldehyde with C6-PdNP in water after 6 h (extracted with ethyl acetate and then passed through a pipette filled with silica gel).

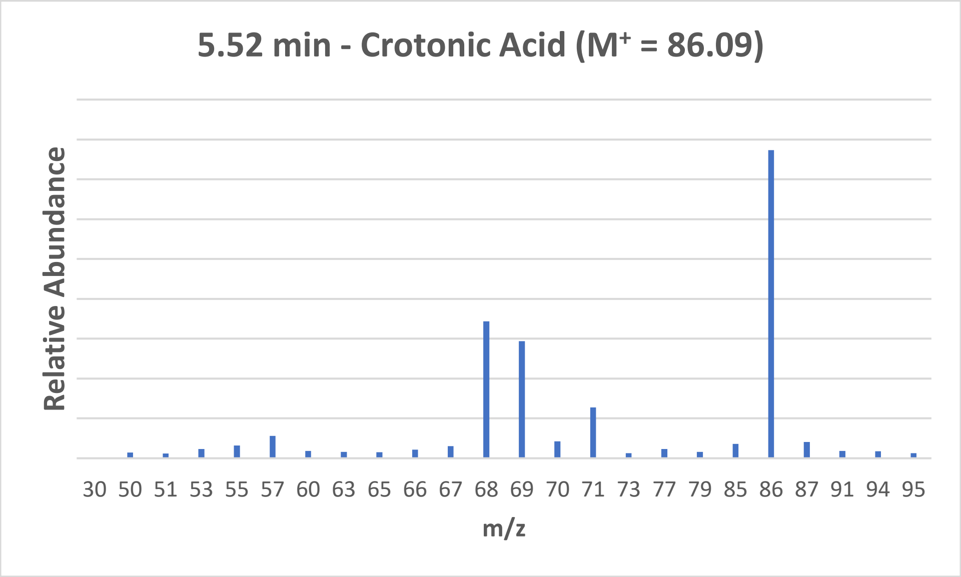

Figure S12. Mass spectrum of the broad peak at 5.31 – 5.59 min corresponds to crotonic acid.

References

1. Gavia, D. J.; Maung, M. S.; Shon, Y.-S. Water-Soluble Pd Nanoparticles Synthesized from ω-Carboxyl-S-Alkanethiosulfate Ligand Precursors as Unimolecular Micelle Catalysts. *ACS Applied Materials & Interfaces* **2013**, 5 (23), 12432–12440.
2. Avila, E.; Nixarlidis, C.; Shon, Y.-S. Water-Soluble Pd Nanoparticles for the Anti-Markovnikov Oxidation of Allyl Benzene in Water. *Nanomaterials* **2023**, *13* (2), 348.
